# Epidemiology of pediatric facial trauma in Chile: A retrospective study of 7,617 cases in 3 years

**DOI:** 10.4317/medoral.19035

**Published:** 2013-08-29

**Authors:** Carolina Collao-González, Alonso Carrasco-Labra, Hsiao H. Sung-Hsieh, Juan Cortés-Araya

**Affiliations:** 1 BSc, DDS, MSc, Oral and Maxillofacial Surgery, Faculty of Dentistry, University of Chile, Department of Public Health, Pontifical Catholic University of Chile; 2 DDS, MSc, PhD(c), Department of Clinical Epidemiology & Biostatistics, McMaster University, Ontario, Canada, Lecturer, Department of Oral and Maxillofacial Surgery, Faculty of Dentistry, Universidad de Chile; 3 DDS, MSc, Clinical Lecturer, Oral and Maxillofacial Surgery, University of Michigan, Ann Arbor, MI, USA, Assistant Professor, Oral Maxillofacial Surgery, Faculty of Dentistry, Universidad de Chile; 4 DDs, OMFS, Full Professor, Department of Oral and Maxillofacial Surgery, University of Chile, and Academic Committee on Admissions Member for the graduate program in Oral and Maxillofacial Surgery, University of Chile

## Abstract

Objectives: To describe the epidemiology of facial trauma injuries in a group of Chilean children aged 15 years or less. Study Design: Retrospective study of case series. Between 2006 and 2009, clinical records of 293,090 patients were reviewed. Data of patients with trauma injuries to the face were collected and evaluated for: age, sex, day and month of hospital admission, cause of injury, anatomical location, type of injury and presence of associated injuries. Results: A total of 7,617 patients with 8,944 injuries were found. Boy to girl ratio was 1,7:1. Preschool age children were most frequently affected. Main cause of injury were falls, soft tissue injuries the most common type of injury. Associated injuries occurred in 11% of cases. Conclusions: Facial trauma presents a significant frequency in the group of Chilean children studied. Preeschool age boys were prone to present facial trauma of mild severity associated to falls.

** Key words:**Facial trauma, pediatric trauma, epidemiology, pediatrics.

## Introduction

Trauma is the leading cause of morbidity and mortality in the pediatric population worldwide (1-4), and Chile is not different in this aspect (5). Out of these injuries, craniofacial trauma is a significant cause of morbidity in the pediatric population. Although children are prone to suffer head and face trauma, international studies reveal that facial trauma has a lower incidence in children compared to the adult population (1-4,6-8). The incidence of pediatric facial fractures range between 1% and 14.7% for victims under the age of 16 and 0.87% to 1% for those younger than 5 years of age (3,4).

Children present unique features that make them particularly prone to sustain head and facial trauma. They have a high head-to-body mass ratio, which results in higher energy impacts to the craniofacial region (4). At birth, the ratio between cranial volume and facial volume is approximately 8:1, which becomes close to 2.5:1 in adults. This retruded position of the face compared to the skull is an important feature that explains the lower incidence of midface and mandibular fractures and higher incidence of cranial injuries in children fewer than 5 years of age. Also, in children younger than 5 years of age the sensory and motor control systems are in development, which increases the frequency of falls and accidental trauma (9). With facial growth following a downward and forward direction, this situation reverses as the child age increases (7,10,11).

Previous retrospective studies reveal that facial trauma has a higher incidence in boys compared to girls, and the primary causes are falls, violence and motor vehicle accidents, whose order varies according to the geographic region under observation (1-4,6,12,13). Soft tissue injuries are more frequent than facial fractures and dentoalveolar trauma (3).

An epidemiological description that includes all traumatic facial injuries on pediatric patients does not exist in Chile to date. The collection and analysis of data regarding this subject is crucial to determine the human and economic costs that facial trauma cause on the patients, the health care system, and for the design and evaluation of preventive measures. Although efforts have been made in Chile generating epidemiological profiles on specific types of traumatic lesions, they do not consider a significant number of individuals and focus only in a specific type of injury (14-16). The aim of this retrospective observational study is to describe the epidemiological characteristics of facial injuries in a group of Chilean children under 15 years of age. The conclusions obtained by this study should be useful for assessing the impact of this type of injury in the pediatric population and the development of preventive measures.

## Material and Methods

The Exequiel González Cortés Children’s Hospital (HEGC), located in the south area of Santiago de Chile, is one of the four public children’s hospitals in the metropolitan area. It covers an approximate population of 1,158,335 habitants. In the 2001 to 2007 period, the HEGC occupied the third place regarding the total number of consultants in the south area. This hospital counts with an Emergency Room (Unidad de Emergencia Hospitalaria or UEH) where this study was carried.

A retrospective study of case series was performed. Clinical records of 293,090 patients less than 15 years of age admitted to the UEH from May 2006 to April 2009 were reviewed. Data of patients with one or more injuries to the face resulting from trauma were retrieved. Patients admitted for follow-up evaluation, burn lesions or isolated fractures of frontal bone, cranial vault or cranial base were excluded. In the selected records the following parameters were evaluated: age, sex, day and month of hospital admission, cause of injury, anatomical location, type of injury and presence of associated injuries.

Patients were divided according to age in three groups: 0 to 5 years old or preschool-age; 6 to 12 years old or school age; and 13 to 15 years old or adolescents. Cause of injury was divided in eight categories: (1) falls; (2) motor vehicle accidents; (3) violence; (4) sports; (5) domestic accidents; (6) animal bites; (7) gunshot wounds; and (8) others. Three types of injury were defined: soft-tissue injuries, bone fractures and dentoalveolar trauma. Location of the soft tissue injuries was registered according to the esthetics facial units described by Fattahi (17). The bone fractures were classified according to the affected site as: (1) mandibular fractures; (2) maxillary fractures, including Le Fort and hard palate fractures; (3) zygomatic fractures; (4) orbital fractures; and (5) nasal fractures (18-22). Dentoalveolar trauma was categorized according to the simplified Andreassen’s classification (23).

Obtained data were registered and processed in an electronic database developed for this specific purpose in Microsoft Access 2007. Data is presented in simple tables. This study was reviewed and approved by the Universidad de Chile, Faculty of Dentistry Ethics Committee.

## Results

In a three-year period, 293,090 medical consults from patients between 1 month and 15 years of age were registered in the UEH. Out of these, 7,617 were related to facial trauma, representing 2.6% of the total. A total of 8,944 traumatic facial injuries were identified.

-Age and sex distribution

**Figure 1:**
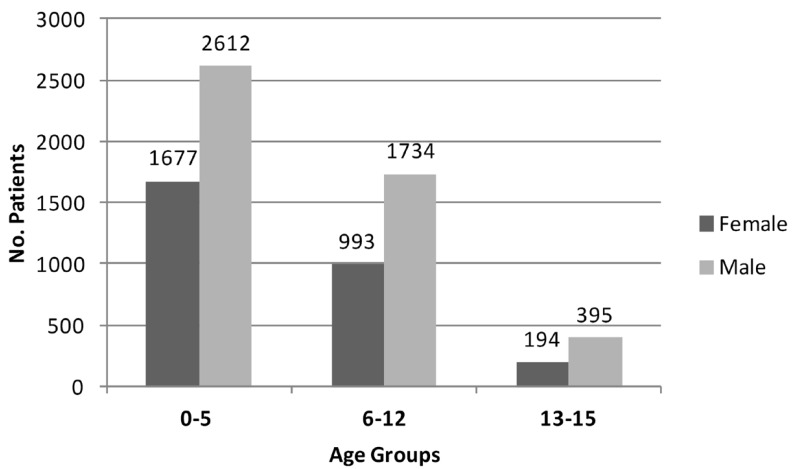
The age and sex distribution at the time of injury of the 7,617 patients with facial trauma treated at the Emergency Unit of Exequiel González Cortés’ Hospital in Santiago de Chile, from 2006 to 2009.

The average age at the moment of the trauma was 5.6 years. Over half of the patients (56.3%) were younger than five years of age, followed by the school age children (35.8%) and adolescents (7.7%). In the yearly breakdown, more facial trauma was reported during the first year of life (16.8%) than any other year (Fig. 1). Most of the children affected were males (62.3%) over females (37.7%), resulting in a male-female ratio of 1.7:1. This difference accentuated as the children grew older, from 1.6:1 in preschoolers to 1.7:1 in school-age and 2:1 in adolescents.

-Temporal distribution

**Figure 2:**
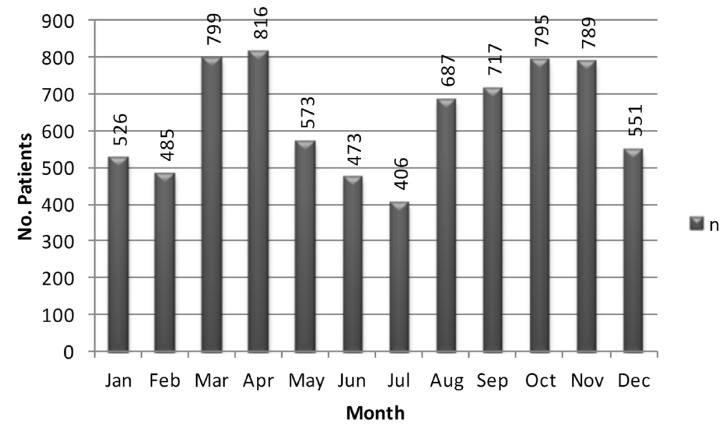
Monthly distribution of injury of the 7,617 patients with facial trauma treated at the emergency unit of the Exe-quiel González Cortés’ Hospital in Santiago de Chile, from 2006 to 2009.

The number of children with facial trauma increased during the observed period, from an average of 199 patients per month in 2006 to 240 patients per month in 2009. The monthly distribution peaked in the spring and summer-fall season, and the minimum registered in the winter season, corresponding to the months of June and July (Fig. 2).

-Etiology

The distribution of causes of injury is shown in  Table 1. The most common causes of injury were falls (53.5%), followed by domestic accidents (28.8%). Combined, these two causes accounted for 82% of the total. Other causes of injury were animal bites, violence and motor vehicle accidents (MVA), the last including motorcycle and pedestrian accidents. The predominance of each cause varies according to the age group: while falls tend to decrease in the older age groups, violence-related trauma increments.

**Table 1:**
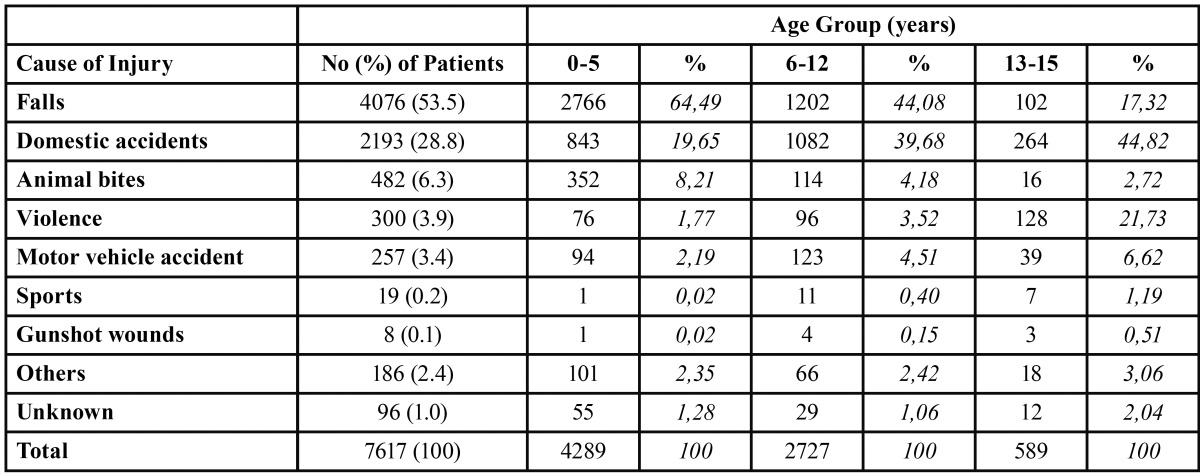
Causes of facial trauma and distribution in age groupsin children with facial trauma.

**Figure 3:**
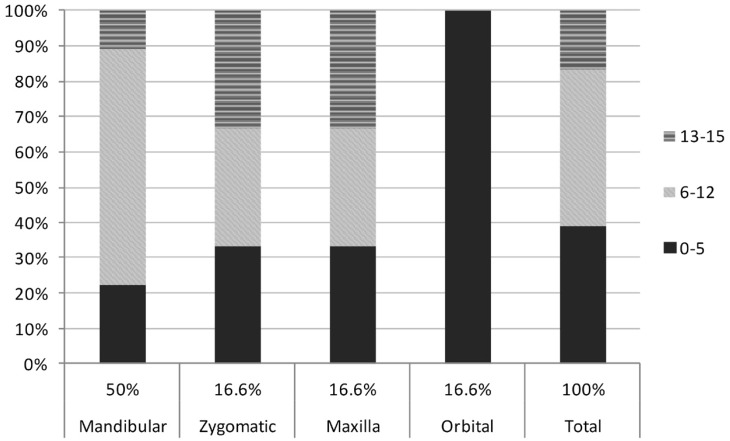
Percent distribution of children with maxillofacial bone fractures by age group in the different anatomical locations.

As to the age distribution, falls and animal bites affected mainly patients under five years of age, while MVA presented predominantly in patients of the 6 to 12 age segment. Violence was an etiology primarily observed in the adolescent patients (Fig. 3).

The distribution of places where the trauma occurred is correspondent with the causes. Most patients suffered trauma at their home (39%, n=2,957) and school (33%, n=2,544). Only 21% of the patients were injured on the street or other public spaces (n=1564).

-Type of injury and localization 

A total of 8,944 facial injuries due to trauma were identified. Out of these, 8,008 (90%) were soft tissue injuries. A 6% of the injuries (n=496) were facial bone fractures and 5% (n=440) dentoalveolar trauma.

Most of the soft tissue injuries were facial lacerations, followed by contusions and abrasions ( Table 2). Only 8% (n=641) affected the intraoral region. The remaining 7,367 injuries that involved facial soft tissue were more often found in the forehead region (34%), followed by the nasal (16%) cheek (10.5%), upper lip (10.1%) and mental (6.7%) regions.

**Table 2:**
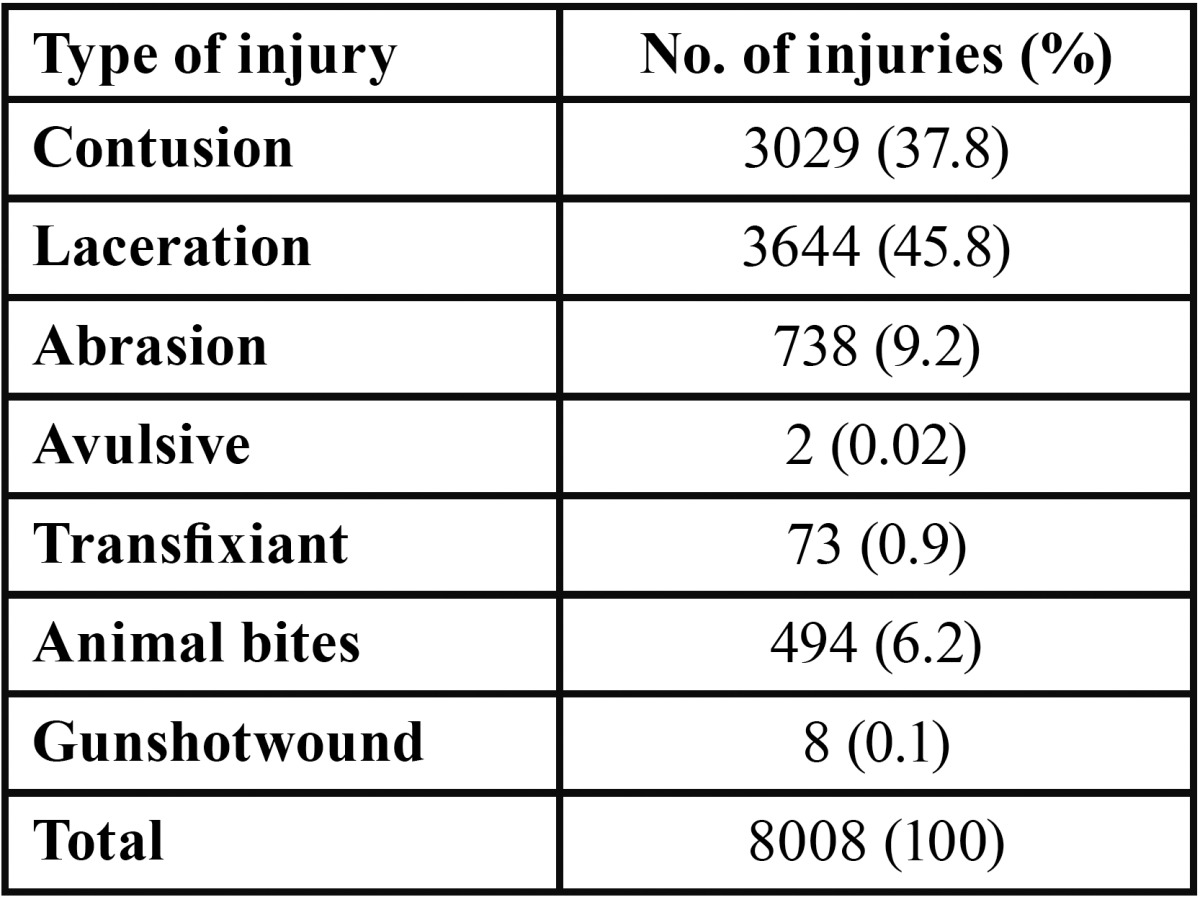
Traumatic facial soft tissue injuries sustained by children.

There were 496 facial bone fractures in 496 patients ( Table 3). Out of these, 96.4% were nasal fractures (n=478), followed by mandible fractures (1.8%, n=9). Fractures of the midface accounted for 1.2% of the cases, including even cases of zygomatic and maxillary fractures (0.6%, n=3 each). Orbital fractures were also observed in 3 patients (0.6%). The distribution of the maxillofacial fractures according to anatomical location can be seen in figure 3. The etiology of these fractures were falls and domestic accidents (77.8%), followed by violence, motor vehicle accident, gunshot and animal bite, each one in equal proportions (5.6%).

**Table 3:**
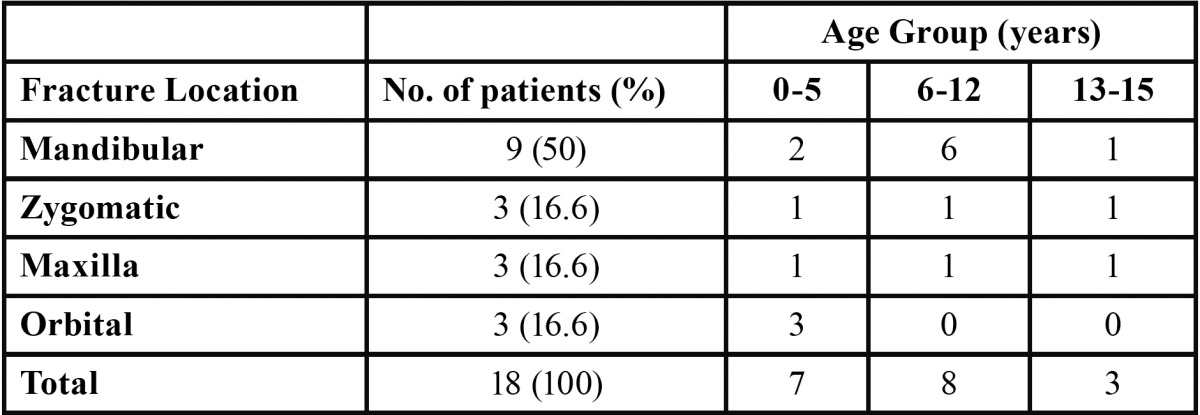
Maxillofacial bone fractures sustained by children.

On dentoalveolar trauma, 440 dentoalveolar injuries in 428 patients were observed. Among the dentoalveolar injuries, 65% were located in the maxillary incisors and 8% in the mandibular incisors; although in a large number of cases (27%) the location was not registered ( Table 4).

**Table 4:**
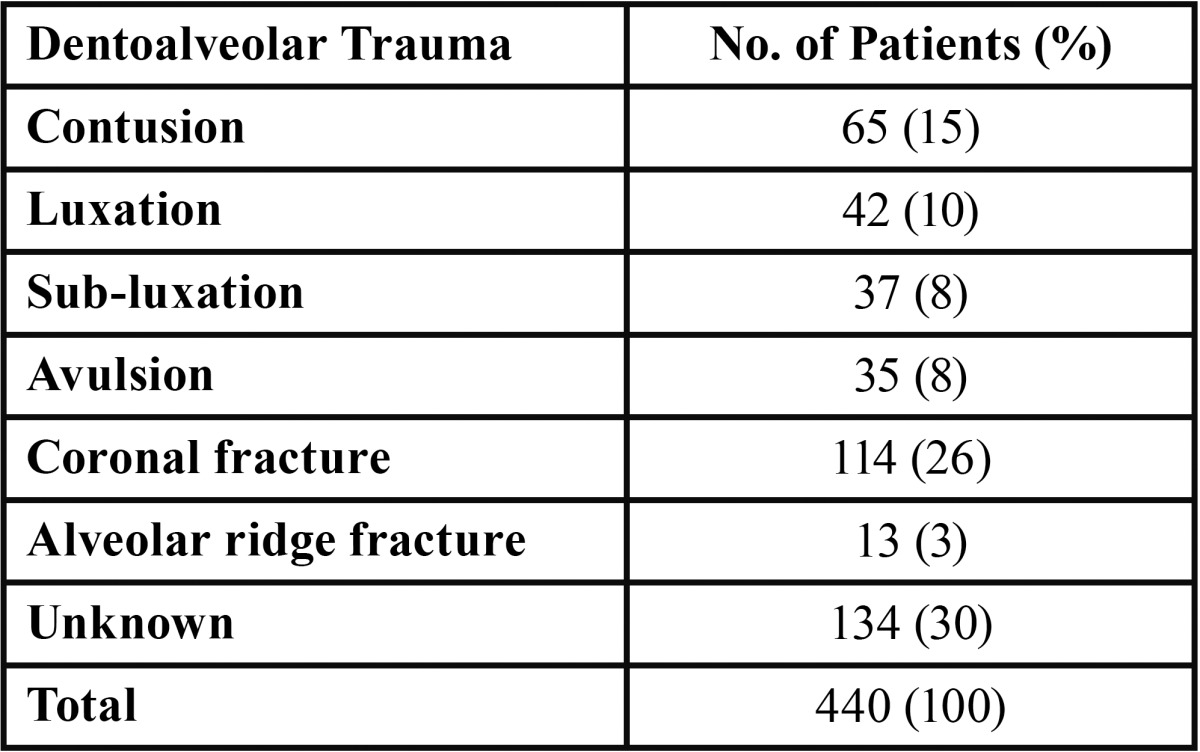
Dentoalveolar trauma sustained by children.

-Associated injuries

Out of the patients with facial traumatic injuries, 11% (n=803) had other associated injuries. The most common associated injuries were to the head and neck (n=431), followed by extremities (n=189) and multiple contusions (n=156). Lesions to the thorax (n=19) and abdomen (n=8) were the least frequent. Among the head/neck injuries, 37.8% were mild head injuries while 37.4% suffered traumatic eye injury and 21% reported traumatic head injury.

## Discussion

Epidemiological studies on facial trauma are diverse regarding inclusion criteria for patients and injuries considered, and their results also vary according to factors like geographic location, socioeconomic status and cultural environment. Most surveys on pediatric facial trauma tend to focus on facial fractures, ignoring other lesions like soft tissue injuries and dental trauma, as the study of Medina et al, who found a frequency of 2.3% of facial fractures in pediatric patients in Chile (13) but does not report frequencies for other maxillofacial injuries. The present study considers all three types of traumatic facial injuries independent of their severity, in a three-year period, being thus the first massive epidemiological profile on facial trauma in Chilean children to date.

The number of patients included in this study is significant, with 7,617 patients that met the inclusion criteria. This represents an average of 7 children per day, seven times higher than what was observed by Gassner et al in a similar study (3). The observed seasonal cyclic behavior was expected, and is associated to a cyclic increase in physical activities in the longer summer days, in contrast to the general inactivity of winter days (3,12,21). The decrease in the frequency of facial trauma registered in the middle of summer is coincident with the school holidays period, when most families travel on vacations (23).

The overall male to female ratio observed, 1.7:1, was similar to the 2:1 reported by others studies (1-4,5,8,12,21,23). The increased frequency of facial trauma in preschool-age children has been reported in studies that include both minor and major injuries (3,7,8,12,24-26), but when considering only major lesions,facial trauma increases in older children (5,6,21,23). This suggests an increased risk for preschool-age children to sustain minor facial trauma. The less marked male to female ratio in these patients versus older ones indicate that such a risk depends on age-related factors that are independent of gender, as the lack of coordination and other motor skills (7,24,25). The main causes of facial trauma were falls and domestic accidents. The preponderance of falls as a cause of trauma in young children has been reported by several different studies, associated to factors as motor development and the typical child behavior (3,7,8,12,24,25). This differs from other reports that describe motor vehicle accidents (MVA) (2-4,5,23,26) and violence (1) as main causes. In this study, only 3.4% of injuries were related to MVA’s, which may be caused by intrinsic variations between countries or the observed sample, and corresponds with the low frequency of facial fractures observed, since these are usually associated with high-energy impacts. Dogbites also affected mainly children under five years of age, who are prone to suffer bite wounds in the head, face and neck (27-29). A 68% of the bites occurred at home, fact that supports the notion that most of children are attacked by the family dog, in the presence of their parents, which is to be considered when designing preventive measures (30).

In consistency with other studies most of the injuries involved soft tissues of the face (3,25,27). These were distributed in a T-shaped zone formed by the forehead, nasal, lip and mental regions, which has been called falling zone in reference to the most common cause of injury in children (24,25). Due to the larger exposition of the face versus the oral cavity, facial injuries were more frequent than oral traumatic lesions (27). In children facial lacerations are commonly associated with slips/trips against hard or angulated surfaces, which make the padding of house furniture an effective preventive measure (8). The lack of this type of preventive policies in Chile could explain the high frequency of lacerations over contusions observed.

Only 496 children suffered from facial bone fractures. This represents a frequency of 6%, which is slightly lower than the 10% reported by Gassner et al, and consisted mainly in nasal bones fractures, which supports previous results that points nasal fractures as the most common facial fracture in children (4,8,11,23,25). This has been attributed to the prominent position of the nose in the face especially in older children and adolescents, and is consistent with the high frequency of falls and domestic accidents observed, as these usually involve low-energy impacts appropriate to cause nasal fractures.

Most of the studies on facial trauma do not include nasal fractures (3,4), possibly for considering them belonging to the otolaryngology field,or had different inclusion criteria regarding severity of injury (1,5,21). When excluding them, mandible fractures were the most common facial fracture in children,agreeing with previous studies that report the mandible as the most affected site (1,3,4,5,9,10,21,23). Mandibular condyle is the most compromised structure in pediatric facial fractures (6,19-21,25), as in the results obtained. The high propensity of pediatric condyle to fracture could be attributed to the high vascularization of the structure and a thin neck that has low resistance to impact forces. Condylar fractures in children present a risk of severe complications as facial growth disturbances, reported in 15% of cases and more frequent in school age children 6, and temporomandibular joint ankylosis, reported in 1-7% of condylar fractures and affecting primarily children younger than five years of age (6). These complications make prevention and early diagnose of mandibular fractures an important issue in pediatric health care.

Midface, orbital and zygomatic complex fractures had an evenly distribution and low number of cases each, similar to literature reports of a frequency in children from 7% to 41% for zigomatic, 1.2% to 20% for maxillary fractures 4 and a 3% to 45% for orbital fractures (22). These numbers could be attributed to the retruded position of the midface, lack of development of the ma-xillary sinuses and lack of prominence of the zygomatic arch, among others.

Children who suffered facial fractures, excluding nasal fractures, had an average age of 7 years. The male to female ratio was 3.5:1, which is considerably higher than the ratio for pediatric facial trauma in general and supports the notion than boys are more prone to suffer from serious injuries than girls.

The frequency of dento alveolar trauma was low in comparison to previous national data reported by Díaz et al, 37.9% in the same age band (15). Since the study is based in hospital encounters, it is most possible that these represent only a small part of all traumatic dental injuries in children. It has been reported that the majority of this type of cases are treated in the community or not treated at all, since patients with this type of injury tend not to go to an urban hospital for treatment (12).

Long term collection of epidemiological data on facial trauma in children is fundamental to make an assessment of the impact of these injuries on the patients and the health care system, and to aid in the design and evaluation of preventive measures. This work is relevant as the first study to provide comprehensive data on the subject in Chile. However, presents limitations in its epidemiological interpretation, as it is based on patients of an urban hospital, which is not necessarily representative of the general pediatric population of Chile.

The results of this study demonstrate that facial trauma is common in children and the frequency of these injuries tends to increase, in consent with reported studies in pointing male children, preschool age and falls as most commonly associated factors with pediatric facial trauma. Future studies are necessary to attain a more representative epidemiological profile of facial trauma in the pediatric population.
